# Anatomical Variations of Anterior Ethmoidal Foramen and Cribriform Plate: Relations With Sex

**DOI:** 10.1097/SCS.0000000000007789

**Published:** 2021-07-15

**Authors:** Daniele Gibelli, Michaela Cellina, Stefano Gibelli, Chiara Floridi, Giovanni Termine, Chiarella Sforza

**Affiliations:** ∗ Department of Biomedical Sciences for Health, University of Milan; † Unit of Radiology, Fatebenefratelli Hospital, ASST Fatebenefratelli Sacco; ‡ Unit of Otolaryngology, Fatebenefratelli Hospital, ASST Fatebenefratelli Sacco, Milan; § Department of Radiology, University Hospital “Umberto I – Lancisi – Salesi,” Ancona, Italy.

**Keywords:** Anterior ethmoidal foramen (AEF), cribriform plate, CT-scan, endoscopic sinus surgery (ESS)

## Abstract

Position of anterior ethmoidal artery and height of lateral lamella of cribriform plate (LLCP) represent critical variants in endoscopy.

In 200 maxillofacial computed tomography scans of patients the position of anterior ethmoidal foramen (AEF) in relation to ethmoid roof was recorded. The height of LLCP was measured and classified according to Keros classification.

Differences in AEF position and distribution of Keros types, and LLCP height according to sex were assessed through chi-square test (*P* < 0.05) and 1-way analysis of covariance (ANCOVA) test, respectively (*P* < 0.05). Differences in LLCP height according to different AEF position were assessed through Mann–Whitney test (*P* < 0.05).

No differences were observed in prevalence of AEF exposure according to sex (*P* > 0.05); LLCP height was higher in males than in females, with a higher frequency of Keros type 3 (*P* < 0.05). Moreover, subjects with AEF exposure had a significantly higher LLCP height (*P* < 0.01).

Results highlighted innovative data useful for improving the knowledge of these sensitive variants.

## Abstract 2

Supplemental Digital Content is available in the text

In the last decades the use of endoscopic surgery of sphenoid and frontal sinuses and ethmoid cells has dramatically increased fields of applications. However, they may be affected by the risk of involving orbits and anterior cranial base during the surgical procedures. The most critical structure is the anterior ethmoidal artery (AEA) passing within the anterior ethmoidal foramen (AEF) and the lateral lamella of the cribriform plate (LLCP).

The AEA usually runs closely apposed or embedded into the skull base; however, it may course freely below the cranial base, surrounded by pneumatized spaces of the ethmoid bone, and linked to the skull base through a bony mesentery.^1^,^2^ In this case the risk of injuring AEA during endoscopic surgical procedures increases. Therefore, the localization of AEF is crucial to avoid possible injuries of AEA.

The LLCP in the ethmoid bone is another critical structure in endoscopic surgery. The LLCP is located at the insertion of fovea ethmoidalis at the skull base and represents the thinnest bone area of the skull base (Fig. 1A). Unfortunately, LLCP is most likely to be damaged during endoscopic surgery. In addition, the height of the LLCP may be very variable with consequent variations in depth of the cribriform plate: in case of deep cribriform plates, the risk of damaging LLCP increases, resulting in the accidental communication between the anterior cranial base and the ethmoidal cells, with cerebrospinal fluid leakage and increased risk of intracranial infections.^3^

**FIGURE 1:**
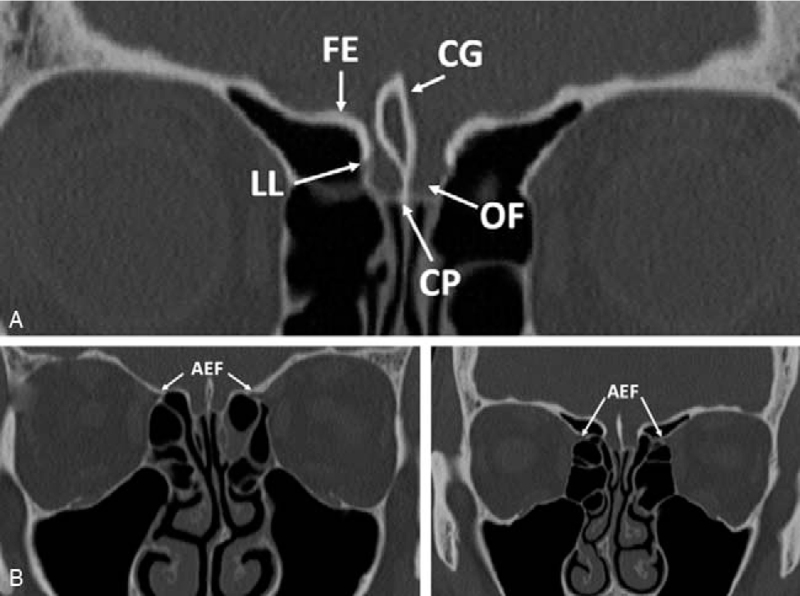
(A) Anatomy of the ethmoid bone. (B) Different positions of the anterior ethmoidal foramen (AEF): on the left, close to the ethmoid roof; on the right, below the ethmoid roof. CG, crista galli; CP, cribriform plate; FE, fovea ethmoidalis; LL, lateral lamella of the cribriform plate; OF, olfactory fossa.

Literature has analyzed the prevalence of the above-mentioned variants in general population.^1^,^4^ However, very little is known about their distribution according to sex; moreover, no study has verified the sexual dimorphism of these variants in relation to general cranial size through a covariate analysis.

This study aims at assessing the position of AEF and the height of LLCP in a sample of computed tomography (CT) scan, analyzing the possible relationships of sex.

## MATERIALS AND METHODS

Two hundred maxillofacial CT scans of patients, equally divided between males (age: 55.1 ± 17.5 years) and females (age: 54.7 ± 19.1 years), were retrospectively assessed. Differences of age according to sex were assessed through Student *t*-test (*P* < 0.05).

All the CT scans were performed through a second-generation dual-source scanner. Somatom Definition Flash (Siemens, Forchheim, Germany; kV: 120, mAs: 320, collimation: 40 × 0.6 mm, tube rotation: 1 second; reconstruction thickness: 3 mm; reconstruction filters: H21s smooth for soft tissues and H60 sharp for bone). Subjects affected by acquired or congenital pathologies involving the cranium were excluded from the study. The study follows international laws and guidelines (Helsinki Declaration) and was approved by the local ethical committee (7331/2019).

The position of AEF in relation to ethmoid roof was recorded, distinguishing if it was located within or close to the bone plate, or if it was below the ethmoid roof (Fig. 1B). The height of LLCP was measured as the distance between the highest point of LLCP and the transversal plane passing through the cribriform plate.

In addition, the height of LLCP was classified according to Keros classification: type 1 between 1 and 3 mm; type 2 between 4 and 7 mm; type 3 between 8 and 16 mm.^5^

In addition, 3 cranial measurements were manually measured on the 3 axes, and in detail: distance between anterior and posterior nasal spine, upper facial height (nasion-prosthion), and biorbital breadth (ectoconchion-ectoconchion distance).

Differences in AEF position and distribution of Keros types according to sex were assessed through chi-square test (*P* < 0.05). Differences in height of LLCP according to sex were assessed through 1-way ANCOVA test using each cranial measurement as covariate (*P* < 0.05).

Differences in height of LLCP between subjects with AEF within or close to ethmoid roof and subjects with AEF located below the ethmoid roof were assessed through Mann–Whitney test (*P* < 0.05).

All the statistical analyses were performed through SPSS software (IBM, USA).

## RESULTS

No statistically significant age differences were found between males and females (*P* < 0.05).

No statistically significant differences were observed in prevalence of AEF position according to sex, for the right (chi-squared: 1.288; *P*: 0.256) or the left side (chi-squared: 3.388; *P*: 0.066).

LLCP height was significantly higher in males (mean: 6.0 ± 2.0 and 6.2 ± 2.1 mm for the right and left side, respectively) than in females (mean: 5.4 ± 1.7 and 5.5 ± 1.7 mm for the right and left side, respectively), independently from the cranial measurement chosen as covariate (*P* < 0.05, Supplementary Digital Content Table 1, Table 2, http://links.lww.com/SCS/C758).

In addition, although type 2 was prevalent in both sexes (Supplementary Digital Content, Table 3, http://links.lww.com/SCS/C758), a significant difference was found in prevalence of Keros types according to sex, being the type 3 more represented in males than in females (chi-squared: 6.922; *P*: 0.031; chi-squared: 8.922; *P*: 0.012, respectively, for the right and the left side).

Finally, subjects with AEF located below the ethmoid roof had a significantly deeper cribriform plate, both on the right and the left side (*P* < 0.01).

## DISCUSSION

Minor complications in endoscopic surgery of paranasal sinuses occur in up to 15% of procedures and include ecchymosis and limited bleeding (under 500 mL); major complications occur in up to 3% and include important bleeding, cerebrospinal fluid leakage, and orbital lesions.^6^ The analysis of anatomical variants may provide an important help in the management of endoscopic surgery of paranasal sinuses.^7^

Ethmoidal arteries are critical points in surgery of paranasal surgery: previous study has already explored the location of the AEA and posterior ethmoidal artery according to bone orbital references, including the anterior lacrimal crest.^8^,^9^,^10^ Usually AEF is positioned below the orbital roof in 14.3% to 84.0% of cases according to different populations^8^; the present results highlighted a prevalence concordant with previous data (42%–59%).

Another innovative conclusion derives for the analysis of the height of LLCP: the lateral lamella of cribriform plate was on average 1 to 2 mm higher in males than in females. This means that the risk of damaging this thin bone wall during endoscopic surgery should be taken into consideration especially in case of male patients. Interestingly, the highlighted differences in LLCP height between males and females are independent from the cranial size.

This result is confirmed also by the division of the sample according to the Keros classification: although type 2 was prevalent in both sexes, type 3, representing the most dangerous variant of LLCP height, was significantly more frequent in males than in females.

In addition, the present study found that LLCP height and position of AEF within the ethmoid cells are strictly related: in details, the longer LLCP height, the more probably AEF is below the ethmoid roof. This conclusion has been already acknowledged in literature by Yenigun et al^4^; however, the lack of significant sexual dimorphism in the frequency of AEF position below the ethmoid roof suggests that other variables may influence this anatomical variant.

## CONCLUSIONS

In conclusion, the present article found that the height of LLCP is significantly higher in males than in females, independently from cranial size, with consequent higher risk of accidental injuries during endoscopic surgery. On the other side, AEF location under the ethmoid roof is similar in both the sexes.

## Supplementary Material
