# Supplementary material for: Anatomical Variations of Anterior Ethmoidal Foramen and Cribriform Plate: Relations With Sex
Source: J Craniofac Surg. 2021 Jul 15;33(1):e2–4. doi: 10.1097/SCS.0000000000007789 (PMC10298176; doi:10.1097/SCS.0000000000007789)
Supplement: Supplementary file 1 [file jcrsu-33-e2-s001.docx]

| LLCP height (mm) | | Mean | SD | Minimum | Maximum |
| --- | --- | --- | --- | --- | --- |
| Right side | Males | 6.0 | 2.0 | 0.7 | 10.8 |
|  | Females | 5.4 | 1.7 | 0.8 | 9.4 |
| Left side | Males | 6.2 | 2.1 | 0.8 | 11.9 |
|  | Females | 5.5 | 1.7 | 1.3 | 9.6 |

Supplemental Table 1: lateral lamella of the ethmoid bone (LLCP) height in males and females; all values are expressed in mm

|  |  | ANS-PNS | NP | BZ |
| --- | --- | --- | --- | --- |
| Right side | F | 4.483 | 3.931 | 3.920 |
|  | p | 0.035 | 0.049 | 0.049 |
|  | Eta squared | 0.022 | 0.020 | 0.020 |
| Left side | F | 4.539 | 4.117 | 4.454 |
|  | p | 0.034 | 0.044 | 0.036 |
|  | Eta squared | 0.023 | 0.020 | 0.022 |

Supplemental Table 2: results from the one-way ANCOVA test for the assessment of statistically significant differences in lateral lamella of the ethmoid bone height according to sex (p<0.05): ANS-PNS: anterior-posterior nasal spine distance; NP: nasion-prosthion distance; BZ: bizygomatic breadth

|  | | Keros classification (%) | | | AEA exposure (%) |
| --- | --- | --- | --- | --- | --- |
|  |  | Type 1 | Type 2 | Type 3 |  |
| Right side | Males | 11 | 61 | 28 | 50 |
|  | Females | 14 | 73 | 13 | 42 |
| Left side | Males | 8 | 63 | 29 | 54 |
|  | Females | 11 | 77 | 12 | 59 |

Supplemental Table 3: prevalence of Keros types and anterior ethmoidal artery (AEA) exposure in males and females; all values are expressed in percentage
